# Potential candidacy for thrombolysis among ischemic stroke patients presenting to Khyber Teaching Hospital, Peshawar

**DOI:** 10.12669/pjms.40.1.7717

**Published:** 2024

**Authors:** Aliena Badshah, Iqbal Haider, Durkho Atif, Muhammad Taimoor

**Affiliations:** 1 Aliena Badshah, Department of Medicine, MTI Khyber, Teaching Hospital Peshawar, Pakistan; 2 Iqbal Haider, Department of Medicine, MTI Khyber, Teaching Hospital Peshawar, Pakistan; 3 Durkho Atif, Department of Medicine, MTI Khyber, Teaching Hospital Peshawar, Pakistan; 4 Muhammad Taimoor, INOVA Neurology, University of Virginia, USA

**Keywords:** Ischemic stroke, Thrombolysis, NIH Stroke Scale

## Abstract

**Objectives:**

To determine the number of ischemic stroke patients presenting to a tertiary care hospital that are potential candidates for thrombolysis.

**Methods:**

This need analysis study was conducted on one hundred consecutive ischemic stroke patients during the last quarter of 2022 at the Department of Medicine, Khyber Teaching Hospital, Peshawar. The Arrival National Institute of Health Stroke Scale (NIH Stroke Scale) was calculated for all suspected stroke patients after a detailed history and clinical examination. An urgent non-contrast-enhanced CT scan brain was carried out. Patients were included in the study group after intra-cranial bleed was excluded on imaging.

**Results:**

The majority (43%) of the patients (male and female) fell in the age group 51-60 years. Fifty-nine (59%) patients had hypertension as a co-morbid condition; 49% had diabetes mellitus while the remaining percentages were constituted by other risk factors. Fifty-seven (57%) patients presented with NIH Stroke Scale between 5-15; 25% had NIH Stroke Scale greater than or equal to 21. Forty-one (41%) patients having daytime (8 am to 8 pm) strokes presented within 4.5 hours of stroke onset to the hospital while 31% of patients having nighttime strokes (8 pm to 8 am) presented within 4.5 hours of stroke onset to the hospital.

**Conclusion:**

The majority (72%) of ischemic stroke patients reached the hospital facility within 4.5 hours of stroke onset. These patients can be benefited from thrombolysis leading to improved outcomes if available in that particular health facility.

## INTRODUCTION

Stroke refers to a condition caused by occlusion or rupture of blood vessels that supply the brain. The brain tissue is vulnerable to ischemia of small duration. Cerebral ischemia causes loss of the structural consistency of the affected brain tissue.^1^ It is followed by cerebral edema and that in turn, causes secondary damage by intensified intracranial pressure and mass effect, misplacing the brain tissue. Less than 10% of ischemic strokes are malignant or massive due to the presence of extensive cerebral edema, causing increased intracranial pressure and brain herniation.^2^,^3^ Numerous risk factors contribute to stroke occurrence, divided into modifiable and non-modifiable. Hypertension, diabetes mellitus, atrial fibrillation, smoking, physical inactivity, carotid stenosis, and hyperlipidemia are among the most commonly known risk factors.^4^ Stroke leads to brain cell death by three known mechanisms - excitotoxicity and ionic imbalance, cell death by apoptosis, and oxidative stress.^5^

Stroke is a very common disease worldwide. Its impact is undeniably huge. A research article published in 2000, it was estimated that 4.5 million deaths occur every year due to stroke worldwide. It also stated that one out of four men and one out of five women aged 45 years are expected to have a stroke if they live up to the age of 85. It was also reported in the same paper that by 2023, there will be an absolute increase in the number of patients experiencing the first stroke of about 30% compared with 1983.^6^ From 1990 to 2019, the absolute number of cases augmented significantly, with up to 70% increase in the incidence of stroke, 43% deaths from stroke, and a 102% increase in the prevalence of stroke.^7^ The situation is particularly alarming in underdeveloped countries like Pakistan.

Our present need analysis aims to find out the frequency of acute ischemic stroke patients presenting to a tertiary care hospital that are potential candidates for thrombolysis. This will provide us with a general idea of how quickly we need to bring about an emergency facility of tPA back in the tertiary care hospital in Peshawar for acute strokes. Thrombolytic, especially Streptokinase was tried in acute stroke patients back in the late 1980s in Pakistan, but it was found to be unproductive with a huge rate of hemorrhagic transformations. This was probably due to trial design errors. Other contributing factors to the letdown of this streptokinase in the local context in the 1980-90s included an extended treatment window, the addition of patients with established infarcts on CT scans, failure to control high arterial pressures, a fixed streptokinase dose and concomitant use of anti-thrombotic.^8^

## METHODS

This need analysis study was conducted in the Department of General Medicine at Khyber Teaching Hospital, Peshawar during the last quarter of 2022.

It was conducted on acute ischemic stroke patients who presented to the emergency department KTH Peshawar. For the purpose of convenience, a convenient consecutive sampling technique was utilized. Hundred (100) consecutive patients were recruited into the study to conduct a needs assessment and determine the time window from stroke onset to presentation in Emergency Department in our setup. A study questionnaire with the inculcation of NIHSS was developed and validated for contents with five subject medical experts utilizing Lynn criteria of 0.80 as cutoff. Face validity was performed on 10 residents as pilot who were later excluded from the study. Informed consent was taken from the patient or his / her immediate accompanying family member in cases where the patient was irritable, drowsy, or comatose.

### Ethical Approval

An ethical approval was granted to this research work from hospital ethical review committee through reference number 739/IREB/DME/KMC Peshawar dated 11-10-2022.

The time of stroke onset or last known well (LKW) and mode of transportation to the hospital was enquired from the patients/family members upon arrival at the hospital. The time of the first encounter with emergency department healthcare personnel was also documented. Arrival National Institute of Health Stroke Scale (NIH Stroke Scale) was calculated for all suspected stroke patients after history and clinical examination. An urgent non-contrast-enhanced CT scan brain was carried out. The time lapse between hospital arrival and CT imaging was documented. Patients were included in the study group after intra-cranial bleed was excluded on imaging. Arrival time in the medical ward was then documented. Stroke onset to hospital arrival time was calculated for these patients. Doctors with validated Stroke certifications issued by the National Institute of Health (NIHS) were exclusively responsible for the direct care of these patients

### Statistical Analysis

All the data was recorded on SPSS Version 20 IBM Corp. Released 2011. IBM SPSS Statistics for Windows, Armonk, NY: IBM Corp. and descriptive statistics were used to analyze the variables accordingly.

## RESULTS

**Fig.1:**
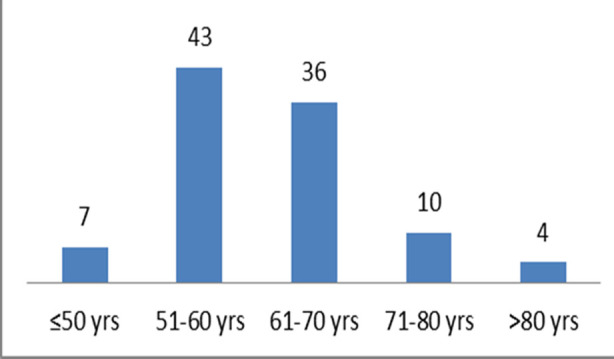
Age distribution

**Fig.2:**
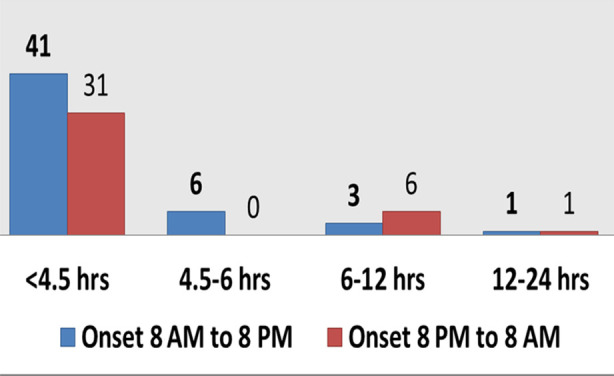
Onset to arrival

The majority (43%) of the patients fell in the age group 51-60 years and 36% of the patients were in the age group 61-70 years (Fig.1). Fifty-nine (59%) patients had hypertension as a co-morbid condition; 49% had diabetes mellitus; 11% had dyslipidemias; 8% had ischemic heart disease; 5% had atrial fibrillation at the time of stroke; 1% history of ischemic stroke and 8% had no known risk factors for stroke. Three (3%) patients presented with an NIHSS of 1-4 (minor stroke); 57 (57%) patients had an NIHSS of 5-15 (moderate stroke); 15 (15%) had an NIHSS of 16-20 (moderate to severe stroke), and 25 (25%) patients had an NIHSS > 21 (severe stroke). Fifty-three (53%) patients had a stroke between 8 am - 8 pm (daytime strokes) while 40% had stroke onset between 8 pm - 8 am (nighttime strokes). Only 7% of patients were having wake-up strokes. Forty-one (41%) daytime (8 am- 8 pm) strokes presented within 4.5 hours of stroke onset to the hospital while 31% of nighttime strokes (8 pm- 8 am) presented within 4.5 hours of stroke onset to the hospital (Fig.2). Eighty-five (85%) patients were brought to the hospital by ambulance whereas 15% were brought by personal vehicle. About 12% of the patients had a non-contrast enhanced CT scan brain performed within 15 minutes of arrival to the accident and emergency department; 31% had it performed within 16-30 minutes of arrival; 17% had it within 31-45 minutes of arrival; 26% had it within 46-60 minutes, and 13% had it after 60 minutes of arrival to the accident and emergency department.

## DISCUSSION

The term stroke refers to the interrupted blood supply to parts of the brain and leads to tissue damage. It can either be acute or chronic but, most strokes present acutely. Ischemic strokes can either be due to thrombosis, embolism, or systemic hypoperfusion, establishing the three subtypes.^9^ The brain tissue is tremendously vulnerable to ischemia of even short duration. Cerebral ischemia causes loss of the structural reliability of the affected brain tissue.^1^ Most of the time, ischemia is followed by cerebral edema and it causes secondary damage by amplified intracranial pressure and mass effect, relocating the brain tissue. Less than 10% of ischemic strokes are malignant or massive due to the presence of extensive cerebral edema, causing increased intracranial pressure and brain herniation.^2^,^3^

The risk factors for stroke occurrence are divided into modifiable and non-modifiable risk factors. Modifiable risk factors include the following: Hypertension, Dyslipidemia, Diabetes Mellitus, smoking, and physical inactivity. Unmodifiable risk factors include the following: Age, race and ethnicity, gender, family history, and genetic disorders. Stroke is the third most common cause of disability and the second most common cause of death worldwide. Globally, ischemia makes up 62.4%, intracerebral hemorrhage 27.9%, and subarachnoid hemorrhage 9.7% of all strokes.^10^

Multiple sources reported that the global 30-day case fatality rate after the first ischemic stroke ranges from 16% to 23%.^11^ China has the greatest contribution to ischemic stroke occurrence worldwide with a mortality rate of up to 115 per 100,000 person-years.^12^ The quality of life of surviving patients is affected leading to financial burdens. Effects from an ischemic stroke can be evaluated with the modified Rankin Scale and the Barthel Index. Unfortunately, not much data has been recorded about the stroke prevalence in Pakistan however, the very first study with 545 patients demonstrated a distressing high lifetime prevalence of cerebrovascular disease in Pakistan, with a total of 119 patients experiencing a stroke.^13^

The prevalence of stroke is 1.2% in the province of KP.^14^ For diagnosing stroke, a detailed history and examination are required along with a neurological assessment which is done through the National Institute of Health Stroke Scale (NIHSS). Urgent brain imaging with either CT or MRI is a mandatory workup. Based on the pathophysiology of ischemic stroke, the treatment aims to restore the blood supply through the thrombosed artery. The only appropriate drug for thrombolysis is intravenous alteplase. Thrombolytic therapy can be used alone or in combination with endovascular therapy for appropriately selected patients with large vessel occlusion. 15

There is always a risk of bleeding with the administration of alteplase and therefore, some physicians are reluctant to its utilization. There is also another fibrinolytic agent, Tenecteplase. According to a 2022 systematic review regarding Tenecteplase versus Alteplase, it was concluded that Tenecteplase is a better option for acute ischemic stroke.^16^ However, the high cost of tenecteplase in comparison to alteplase is the main hindrance in resource-limited countries. Another meta-analysis in 2011 documented that intravenous tissue plasminogen activators reduced three-month mortality in patients >80 years.^17^ Thrombolysis using alteplase is usually recommended when a patient presents within 4.5 hours of the onset of stroke symptoms and intracranial hemorrhage has been excluded through imaging.^18^ Intravenous alteplase convalesces functional consequences at three to six months when given within 4.5 hours of ischemic stroke onset.^19^ However, these drugs cannot be used freely without bearing the brunt of its side effects. Therefore, there are guidelines for their uses, stating a strict perimeter of indications and contraindications including absolute and relative.^20^

If the patient presents within 4.5 hours of the onset of symptoms and is eligible (meeting the criteria for thrombolysis), Intravenous thrombolysis is the first line of management. Another method, Mechanical thrombectomy, is offered to appropriate patients with large vessel occlusion. A meta-analysis from 2011 highlighted that intravenous tissue plasminogen activators reduce three-month mortality in patients aged >80 years.^17^ Alteplase improves outcomes at three to six months when administered within the 4.5 hours window.^21^ Tenecteplase, another substitute for alteplase, is considered better than alteplase with excellent functional outcomes.^22^

In the United States, a retrospective study was done among 1026 ischemic stroke patients in which 513 were treated with tPA and 513 were not; It concluded that those treated with intravenous tPA experienced long-term clinical advantages in survival and functional status.^23^ Another cohort study with five years follow-up (1317 patients treated with intravenous thrombolysis and 1317 patients not treated with intravenous thrombolysis) concluded in 2021 that thrombolysis in acute ischemic stroke reduced short-term mortality by 47% and did not significantly reduce long-term mortality.^24^

This study conducted in Khyber Teaching Hospital, Peshawar helped to quantify the number of patients that could benefit from the administration of thrombolytics. Eighty six percent of the stroke population had received a non-contrast enhanced CT scan within the first hour of presentation to Accident and Emergency department. This duration can be further improved if a separate CT scanner is installed for stroke patients. This will improve channelization of stroke patients for CT scan with better time lapse data between hospital arrival and arrival to CT brain. This study also concluded that 72% of ischemic stroke patients reached the hospital facility within 4.5 hours of stroke onset. Had they received the thrombolytics then, they could have had a high probability to restore blood supply preventing further injury to brain tissue. Consequently, we would have more patients restoring to normal lives from strokes. This need-analysis study nullifies the traditional mindset in our healthcare setup that our stroke patients are not brought to the emergency department in this life-saving window period of 4.5 hours. This data was shared with the concerned stakeholders and they were convinced to arrange IV Alteplase in three public sector hospitals of Peshawar free of cost for the qualifying patients of acute ischemic stroke. Two of three MTI Public sector hospitals in Peshawar have initiated this IV tPA therapy for needy patients in the first week of 2023.

### Limitation

A single-centered trial with a limited number of patients is the major limitation of this research work. However, being a need assessment study of a pilot nature, the findings are highly favorable and have a positive impact in practice changing attitudes among all stakeholders from the local perspective.

## CONCLUSION

The majority of acute ischemic stroke patients reached the tertiary care hospital facility in our setup within 4.5 hours of the onset of ischemic stroke. This means they can benefit from thrombolysis and so their stroke outcomes can improve. We need to take further steps to implement the use of thrombolytics in the emergency department in tertiary care hospitals of Peshawar.

### Authors’ Contribution:

**AB:** Concept, Data collection, Compilation of results, formatting of the article, responsible for accuracy and integrity of the work. **IH:** Concept, Study design, Data analysis, Critical appraisal, and Discussion Writing. **DA:** Overall compilation of the article, Proofreading, Review.**MT:** Data compilation, critical review, Proofreading, Bibliography. All authors contributed as per authorship’s criteria of ICMJE and are accountable for each step of this manuscript.
